# Design of the POINT study: Pharmacotherapy Optimisation through Integration of a Non-dispensing pharmacist in a primary care Team (POINT)

**DOI:** 10.1186/s12875-015-0296-8

**Published:** 2015-07-02

**Authors:** Ankie C.M. Hazen, Vivianne M. Sloeserwij, Dorien L.M. Zwart, Antoinette A. de Bont, Marcel L. Bouvy, Johan J. de Gier, Niek J. de Wit, Anne J. Leendertse

**Affiliations:** Department of General Practice, Julius Centre for Health Sciences and Primary Care, University Medical Centre Utrecht, Utrecht, The Netherlands; Institute of Health Policy and Management, Erasmus University Rotterdam, Rotterdam, The Netherlands; Department of Pharmaceutical Sciences, Utrecht University, Utrecht, The Netherlands; Department of Pharmacotherapy and Pharmaceutical Care, University of Groningen, Groningen, The Netherlands

**Keywords:** Pharmacotherapy, Polypharmacy, Non-dispensing clinical pharmacist, General practice, Primary care, Hospitalisation

## Abstract

**Background:**

In the Netherlands, 5.6 % of acute hospital admissions are medication-related. Almost half of these admissions are potentially preventable. Reviewing medication in patients at risk in primary care might prevent these hospital admissions. At present, implementation of medication reviews in primary care is suboptimal: pharmacists lack access to patient information, pharmacists are short of clinical knowledge and skills, and working processes of pharmacists (focus on dispensing) and general practitioners (focus on clinical practice) match poorly. Integration of the pharmacist in the primary health care team might improve pharmaceutical care outcomes.

The aim of this study is to evaluate the effect of integration of a non-dispensing pharmacist in general practice on the safety of pharmacotherapy in the Netherlands.

**Methods:**

The POINT study is a non-randomised controlled intervention study with pre-post comparison in an integrated primary care setting. We compare three different models of pharmaceutical care provision in primary care: 1) a non-dispensing pharmacist as an integral member of a primary care team, 2) a pharmacist in a community pharmacy with a predefined training in performing medication reviews and 3) a pharmacist in a community pharmacy (care as usual). In all models, GPs remain accountable for individual medication prescription. In the first model, ten non-dispensing clinical pharmacists are posted in ten primary care practices (including 5 – 10 000 patients each) for a period of 15 months. These non-dispensing pharmacists perform patient consultations, including medication reviews, and share responsibility for the pharmaceutical care provided in the practice. The two other groups consist of ten primary care practices with collaborating pharmacists. The main outcome measurement is the number of medication-related hospital admissions during follow-up. Secondary outcome measurements are potential medication errors, drug burden index and costs. Parallel to this study, a qualitative study is conducted to evaluate the feasibility of introducing a NDP in general practice.

**Discussion:**

As the POINT study is a large-scale intervention study, it should provide evidence as to whether integration of a non-dispensing clinical pharmacist in primary care will result in safer pharmacotherapy. The qualitative study also generates knowledge on the optimal implementation of this model in primary care. Results are expected in 2016.

**Trial registration number:**

NTR4389, The Netherlands National Trial Register, 07-01-2014.

## Background

Adverse drug events account for 5,6 % of acute hospital admissions in the Netherlands. Almost half of these admissions are potentially preventable [1]. Older age, polypharmacy, multimorbidity, impaired cognition and impaired renal function have been identified as risk factors for these preventable medication-related hospital admissions (HARMs) [1]. Given the ageing of the population, the population at risk will grow in near future. Hence, new strategies are needed to improve the effectiveness and safety of pharmacotherapy in clinical practice and to prevent these hospital admissions.

As most of the pharmacotherapy is initiated in general practice, its quality may be primarily improved by structural reviewing patients’ medication in primary care. So far, the results of studies on the effectiveness of medication reviews have been inconclusive: several studies reported a positive effect on the number of drug therapy problems [2–7], but no effect on morbidity, mortality or quality of life was found.

Several difficulties hamper the implementation of medication reviews in primary care [8–10] and may have contributed to the inconclusiveness of these results. First of all, as community pharmacists get no or an insufficient fee for performing medication reviews, a financial incentive is lacking. However, this does not seem to be the only problem. Another important difficulty in the implementation is the lack of information: community pharmacists do not have access to routine patient records. Consequently, performing proper medication reviews is often impeded, as not all available information can be taken into account. Third, pharmacists lack clinical pharmacology knowledge and clinical reasoning skills, for pharmaceutical training and practice are historically drug product oriented instead of patient oriented. Community pharmacists’ tasks mainly concern the organisation and monitoring of logistic processes (e.g. dispensing the right medication in the right dose to the right patient); community pharmacists perform little to no direct pharmaceutical patient care. As a result, pharmacists have sparse experience in clinical pharmacotherapy. Fourth, in the present system pharmacists and general practitioners (GPs) have different responsibilities, backgrounds and working processes, resulting in inadequate collaboration [11]. Fifth, the present way of practicing of both GPs and pharmacists is mainly reactive, while the pharmaceutical care process requires a proactive approach. Finally, there is a misfit between time-consuming nature of performing medication reviews and the current workload of both GPs and pharmacists.

Implementation of a non-dispensing pharmacist (NDP) in primary care teams might address these implementation problems and improve outcomes of pharmaceutical care. The NDP – as a healthcare team member – would have access to patient records and the required clinical information. The lack of clinical knowledge and skills of the pharmacist could be overcome by a training in clinical pharmacy. Collaboration with the GP is expected to improve, because the NDP is positioned into the clinical practice and is a full member of the primary care team, with the GP as head of the team. Furthermore, as the NDP’s scope alters from drug product oriented to patient oriented, the professional perspective will collide better with that of the GP [12, 13]. Finally, this change in scope relieves the NDP of his responsibility for the dispensing process, and enables the NDP to work fulltime on the improvement of pharmacotherapy.

This model of integrated pharmaceutical care has already been studied in Canada [14, 15], Australia [16] and the United States of America [17]. It was found that the model has the potential to address many of the barriers to effective pharmaceutical care in the ways described above, thereby optimising medication use and hence leading to better healthcare outcomes [14, 16]. In Canada, physicians recognised many interprofessional benefits by working with a pharmacist directly integrated into their practice. Also, benefits of improved education were described [14]. The Australian study reported a significant reduction in medication-related problems after intervention by the pharmacists, and a significant improvement of adherence to the medication regimen [16]. In the USA, both GPs and patients perceived qualitative benefits from the pharmacotherapy consultations [17].

However, the ultimate benefit of this model for patients, namely the prevention of HARMs, has not been demonstrated yet. Therefore, we designed the Pharmacotherapy Optimisation through Integration of a Non-dispensing pharmacist in a primary care Team (POINT) study, in which we assess, amongst others, the effect of a non-dispensing pharmacist on medication-related hospital admissions.

## Methods

### Design

The POINT study is a non-randomised, controlled intervention study with pre-post comparison (see Table 1 for a time schedule of the POINT study). Three different models of pharmaceutical care provision in primary care will be compared:

**Table 1:** Time schedule of the POINT-study

Period	Dates
Pre intervention period (1 year)	1^st^ of January 2013 – 31^st^ of December 2013
Start-up period, prior to intervention period (3 months)	1^st^ of March 2014 – 31^st^ of May 2014
Intervention period (1 year)	1^st^ of June 2014 – 31^st^ of May 2015

- *Group A (intervention group)*: a GP practice with a non-dispensing pharmacist based in the practice as an integral member of the primary healthcare team;
- *Group B (control group 1)*: ‘upgraded’ care as usual: a GP practice collaborating with a dispensing pharmacist based in a community pharmacy in the traditional way, with the pharmacist having had a predefined, certified additional training in reviewing medication,
- *Group C (control group 2)*: care as usual: a GP practice collaborating with a dispensing pharmacist based in a community pharmacy in the traditional way.

**Fig. 1:**
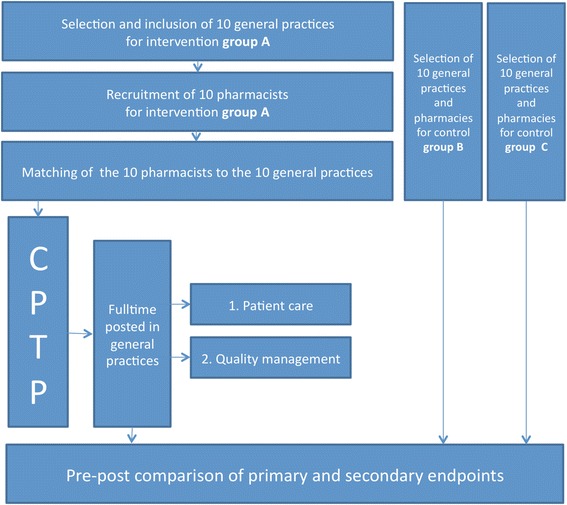
Flowchart of the study design. Abbreviations used: CPTP Clinical Pharmacy Training Program (newly developed for the intervention)

A flowchart of the study design is shown in Fig. 1. Concurrently, a qualitative implementation study is performed.

The protocol was peer-reviewed by the funding organisation.

### Setting

The project is implemented within primary care practices from the Julius General Practitioners Network (University Medical Centre Utrecht) and the Academic Network of General Practitioners (VU University Medical Centre Amsterdam). These networks consist of more than 200 collaborating general practices.

#### Group A: selecting GPs, non-dispensing pharmacists and matching both

General practices from the above mentioned networks are all pro-actively invited to participate in the POINT study. Ten general practices are selected, based on the following criteria: willingness of the GPs to participate in the project; willingness of the GPs to cooperate in the development and evaluation of the role of the NDP; minimum of 5000 registered patients; availability of an office for a NDP, with access to the GP information system; minimum of one practice nurse working on disease management programs for chronic conditions such as chronic obstructive pulmonary disease, diabetes, cardiovascular disease or mental health; healthcare centre accredited by the Dutch College of General Practitioners (NHG) [18]. The research collaboration is formalised in a collaboration agreement.

Ten non-dispensing pharmacists are employed, using a structured application procedure. All participating pharmacists have a master degree in pharmacy (PharmD) and preferably have working experience in providing pharmaceutical care to individual patients. Furthermore, in the selection procedure communication and collaboration skills, as well as pharmacotherapy knowledge, empathy, self-reflection skills and innovative attitude are emphasized.

Subsequently, each NDP is posted in one of the ten selected primary care centres in Utrecht or Amsterdam regions. The NDPs work full time and exclusively in the general practices, for a period of 15 months. The introduction of such a new role in a healthcare practice is complicated and faces a variety of challenges [14]. For example, pharmacists need to be trained to fulfil their new tasks, both pharmacists and GPs have to collaborate closely and GPs have to explore the complementary role of the NDP. Therefore, the first three months are used as a start-up period before actually starting the intervention period.

#### Group B and C: selecting GPs and collaborating pharmacists

**Fig. 2:**
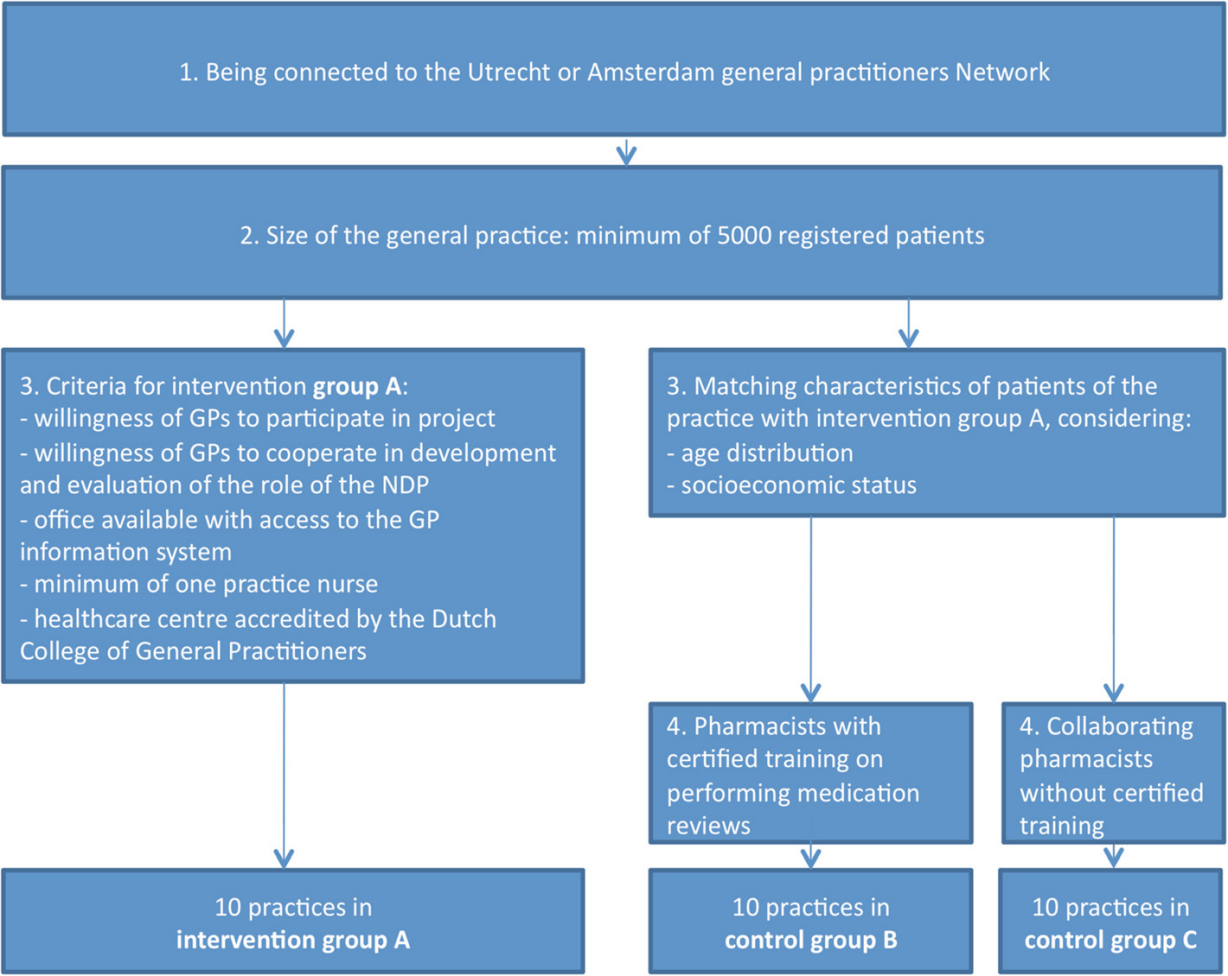
Overview of the selection criteria for general practices for group A, B and C

For both group B and C, ten general practices and collaborating pharmacies are selected from the above-mentioned networks as well. Criteria for participation are comparable to those concerning the size of the practices, described for group A. In addition, characteristics of patients of practices in groups B and C were matched as far as possible with group A, considering age distribution and socioeconomic status. Subsequently, practices and collaborating pharmacies are assigned to group B or C, depending upon whether the collaborating pharmacists have completed a certified training program on performing medication reviews in the Netherlands [19, 20], or not, respectively. See Fig. 2.

### Intervention

To improve the safety of pharmacotherapy within the general practice, the intervention in group A by the NDPs aims at two levels: individual patient consultation and quality management on an organisational level. Herewith, the NDPs are responsible for the medication management and pharmaceutical care provided in the general practice. The NDPs perform complementary work and do not take over tasks of the GP nor the community pharmacist.

### Individual patient consultation

The patient care process consists of an assessment of the patient’s drug-related needs, a care plan to meet the specific needs of the patient, and a follow-up evaluation to determine the impact of the decisions made and actions taken. In practice, the NDP provides pharmaceutical patient care for patients who are considered to be at risk of adverse drug events, such as HARMs. These patients, mostly of older age, with multimorbidity and polypharmacy (chronic use of five or more medicines) [1], are either pro-actively invited by the NDP or referred by the GP to discuss and review their medication. Also, patients can make an appointment for a medication assessment at their own request. During the first consultation, which is preferably a home visit, the NDP will work on a therapeutic relationship and interviews the patient to gather information on the patient’s experiences with and believes about medication, in order to assess his or her drug-related needs. Questions concern the goal of therapy for the patient, the current and past medication history, adherence to the medication regimen and patient reported medication issues. Afterwards, the NDP integrates the patient reported experiences and believes with the medical status to determine whether there are potential drug therapy problems. If necessary, the NDP provides recommendations for optimisation of pharmacotherapy to the GP: suggestions to stop, start or switch medication, to adjust dosages, or for actions to improve adherence. These recommendations result in a documented individual pharmaceutical care plan, as part of the patient’s medical record. The implementation of recommendations is monitored by the NDP. Follow-up contacts can be conducted as a home visit, a practice visit or by telephone.

Furthermore, the NDP covers other aspects of pharmaceutical patient care, such as individual consultations for specific drug therapy problems or questions, and medication reconciliation in patients discharged from hospital.

All patient level interventions involve ongoing on-location collaboration with the healthcare team – being GP, practice nurses, assistants and the community pharmacists. The NDP is available at the GP practice and has daily formal and informal meetings with the GP in order to establish individual pharmaceutical care plans and to report on plans in progress. All members of the healthcare team can easily approach the NDP with questions about medication and patients’ pharmacotherapy.

### Quality management

The NDP aims to improve medication safety on an organisational level, through optimisation of processes within the practice around repeat prescribing, clinical care paths, administrative efficiencies and identification of common medication errors. The NDP is looking for possible optimisation options in medication regimens, such as monitoring renal function and electrolytes with indicated pharmacotherapy, tapering the chronic use of proton pump inhibitors, and optimising antibiotic prescribing. Hereby, the NDP organises targeted programs to improve the quality of pharmaceutical care in the practice. Also, the NDP provides education of patients and professionals involved.

#### Training program group A

To train the NDPs for their new role, a specialised Clinical Pharmacy Training Program (CPTP) is developed, based on workplace learning and the Canadian Medical Education Directions for Specialists (CanMEDS) Roles [21]. The CPTP started with a six-day training workshop, an internship in a nursing home and assignments in practice. Plenary education days are gradually decreased and days in the general practice gradually increased, ending with full time practice work with weekly education days at the university. Key elements of the training are consultation and communication skills, clinical reasoning, clinical pharmacotherapy and being reflective in practice. NDPs are trained to use a patient centred approach in providing care, instead of a drug product centred approach. Barriers to implementation are discussed and ongoing support is provided through structural intervision sessions and a mentorship and buddy program [22].

### Outcomes and measurements

#### Primary outcome: medication-related hospital admissions (HARMs)

The primary outcome is the number of medication-related hospital admissions (HARMs) in the high-risk population. HARMs are defined as hospitalisations related to adverse drug events. To identify these medication-related hospital admissions, two pharmacists with clinical experience will independently assess each hospitalisation that occurred in the study population during follow-up, using discharge information combined with the medical and medication history. They will assess the causal relationship between the suspected medicine and the reason for hospitalisation, according to an adjusted version of the algorithm by Kramer et al. [23]. In this version, three questions need to be answered (in contrast to six questions in the original algorithm): whether the reason for admission is known to be an adverse event of the suspected medicine, whether alternative causes can explain the relationship between the suspected medicine and the adverse event, and whether a plausible time relationship exists between the adverse event and the start of medication administration (or the occurrence of the medication error). On the basis of the answers, causality is classified as “possible”, “probable”, or “unlikely”. Cases with an assessment of unlikely will be excluded.

#### Secondary outcomes

##### Potential medication errors

The percentage of patients with potential medication errors will be measured [24]. These potential medication errors mainly concern prescription errors, such as under- and overprescribing and dosage errors. Other potential medication errors might be due to medication that is not or insufficiently effective, or to inadequate monitoring of the effects of the therapy. Also administration errors, such as non-adherence problems, will be measured as potential medication errors. A complete list of included potential medication errors can be found in Table 2.

**Table 2:** Overview of outcomes, measurements and data sources

Outcomes	Measurement	Data sources
*Primary outcome*		
Frequency of HARMs	Number of HARMs	DL, MH, MED
Secondary outcomes		
Potential medication errors	% patients with:	
	- medication not indicated	MED/MR
	- underprescribing	MED/MR
	- dosing error (too low or too high)	MED/MR
	- therapeutic duplication medication	MED/MR
	- medication contra-indicated	MED/MR
	- drug-drug interactions	MED/MR
	- medication not effective	MED/MR
	- inadequately monitored therapy	MED/MR
	- administration errors (e.g. non-adherence)	MED/MR
Drug burden index	Drug burden of medications with sedative and/or anticholinergic effects	MR
Costs	Medication costs and healthcare-related costs	Database of insurance company

HARM hospital admission related to medication, DL discharge letter, MH medical history (with ICPC codes for diagnoses), MED medication records (including ATC code, dose and strength), MR medical records (including laboratory markers and measurements such as blood pressure, pulse and body mass index)

##### Drug burden index

The drug burden index will be calculated for every patient. This drug burden index measures exposure to anticholinergic and sedative medication, and is associated with poorer physical and cognitive performance in older people [25]. Hence, the drug burden index can be seen as a proxy of drug therapy risk and medication safety.

##### Costs

A cost analysis will be performed, based upon reimbursement data from databases of a Dutch major health insurance company. Direct medical costs, such as for medication, hospital care, specialist care, diagnostic tests and other healthcare-related costs will be included.

### Data collection

Data of all patients in groups A, B and C are accessible through the routine health care databases of the Julius General Practitioners Network (Utrecht) and the Academic Network of General Practitioners (Amsterdam). After the intervention period (see Table 1), key data will be extracted anonymously from the electronic medical records in the general practices of both the pre- and post-intervention period, through standard procedures and existing algorithms. These data (see Table 2) are combined with reimbursement data from the major healthcare insurance company in the Utrecht and Amsterdam region, obtaining 40-55 % of the reimbursement data of the region. No data will be obtained directly from patients.

### Confounding factors

To be able to control for possible confounding, characteristics of the involved general practices and pharmacies in each group will be collected, using a questionnaire. Additional information will be gathered about pharmaceutical care provision, the medication review protocol used, the setting of the pharmacy and the general practice, the collaboration between the pharmacy and the general practice and agreements on pharmaceutical care provision.

### Analyses and statistical method

All primary and secondary outcomes will be compared in pre-post analyses and between groups comparisons will be conducted. Descriptive statistics will be calculated for the baseline characteristics according to data of the overall population in group A, B and C, as well as for the high risk patients. The effect on the primary outcome will be tested with logistic multilevel analysis. The potential medication errors, drug burden index and costs will be tested with mixed effect models. Baseline characteristics can be integrated into the mixed effect models to control for confounding.

### Sample size calculation

With an expected prevalence of 4,5 % HARMs in 12 months within the high-risk population [26], we expect an effect of 50 % reduction of HARMs [1]. To show a statistically significant difference between the intervention group A and control group C, we include ten practices, with a total of 45.000 patients, in each group. As 6,4 % of patients in an average GP practice are part of the high risk elderly population [26], this means that in each arm at least 2850 high risk patients are included. This is based on an alpha of 0,05 and a power (1-beta) of 0,8.

### Qualitative study

In order to assess the feasibility of introducing a NDP in general practice, parallel to the POINT study qualitative data hereon is systematically collected. Semi-structured interviews with participating GPs and NDPs are conducted, and their views are described. Patients who are seen by a NDP are asked about their perceptions and experiences, using anonymised questionnaires. Hereby, conditions that hinder or facilitate the introduction of a NDP in general practice in the Netherlands may be identified.

### Privacy and informed consent/Ethical approval

Based on the Dutch law for patient data protection, this study was exempt of formal medical-ethical approval by the Medical Ethical Committee University Medical Centre Utrecht. (METC protocol number 13-432C).

## Discussion

The POINT study aims to improve safety of pharmacotherapy in primary care, by introducing a non-dispensing pharmacist as a member of the primary care team in the Netherlands. This intervention aims to improve pharmaceutical care at both patient level and organisational level. Therefore, it may be more effective than a singular intervention, such as current medication reviews. A comparison will be made with two existing models of pharmaceutical care provision in primary care. This comparison will demonstrate whether the introduction of the NDP is more effective in improving the quality and safety of pharmacotherapy than existing care models.

Several methodological challenges were faced during the design of the POINT study.

### Choice for the design

Despite the fact that a randomised controlled trial is the preferred design to evaluate the effect of an intervention, we thoughtfully chose to use a non-randomised model. In our opinion, willingness of all participating parties to improve pharmaceutical patient care is a key condition for the implementation of this intervention to succeed. This has been recognised before, during the implementation of a pharmacist in primary care in Canada [14]. Therefore, general practices participating in the intervention group of this study are selected instead of randomly allocated to one of three research arms.

This selection, of course, has disadvantages. Once proven effective, the broad implementation of this new function might be challenging because of the high standards we set for participating practices in this study. In addition, selection of motivated general practices might mask the effect of the intervention. As these practices are motivated to improve pharmaceutical care, standard pharmaceutical care might be better than average beforehand, leaving little room for improvement. By including pre-post analyses, we attempt to obviate this problem.

### Composition of the intervention

The introduction of the NDP is considered a complex intervention. This is for intervening at different care levels, as well as for integrating a new professional into the primary work processes, which requires redistribution of tasks and responsibilities around pharmacotherapeutic care. Although the tasks of the NDP are predefined, the actual implementation in the individual GP practices cannot be protocolled: in order to increase the likelihood of a successful implementation of the intervention, the intervention has to be aligned to the needs of each participating centre. Consequently, the actual implementation of the intervention itself may be heterogeneous. This can blur quantitative measurements. Therefore, parallel to this study, we conduct a qualitative study as described earlier. With this study, we will list facilitators and barriers to the implementation process, in order to assess the feasibility of introducing a NDP in a complex healthcare setting in daily practice.

#### Development of the clinical pharmacy training program

The clinical pharmacy training program (CPTP) has been newly developed for the POINT study and has neither been validated nor accredited. As the CPTP is developed by experts in the field of education, based on the theoretical frameworks of Vermunt, Kolb and Merrienboer [27–29] and as it is embedded in the department of vocational training for general practice, it is expected to be an adequate postgraduate training for the NDPs. Within the context of the POINT intervention study, the program is evaluated and attuned on a structural basis.

### Choice of the primary outcome measurement

In the context of ‘primum non nocere’ [30] the prime aim of this study is to improve the safety of pharmacotherapy. Therefore, we chose reduction of medication-related hospital admissions (HARMs), being a severe adverse drug event, as primary outcome. This choice is, however, challenging in several aspects.

First, the incidence of HARMs in primary care is low. Although 5.6 % of acute hospital admissions are related to medication [1], this accounts for only about 3.4 medication-related hospital admissions per GP on a yearly basis – which means around 12–16 HARMs per participating practice in this study. In addition, we do have a limited follow-up period of only one year. However, our sample size calculation is based upon the occurence of HARMs in a large group, so we expect this problem to be adequately addressed. Last, measuring HARMs is challenging for quite detailed data have to be obtained in order to determine HARMs. Causality assessments in the POINT study will be based upon information of discharge letters, which is limited information. However, using this amount of information to determine HARMs has been done before [31]. Also, we do have experience from previous studies [1, 26] and will use a validated method to identify the primary outcome parameter.

### Availability of data for secondary outcome measurements

To correctly measure and analyse the secondary outcomes, the required data need to be properly documented in the GPs’ information systems. Due to the heterogeneous study setting we are dependent on the diverse working methods of the participating healthcare providers. As this possible loss of information will show equally in each research arm, we expect this will not influence our study results.

The cost evaluation performed in this study will yield an insight in the direct medical costs of each model of pharmaceutical care provision in primary care. For this evaluation, a subgroup of patients will be analysed, as data of the insurance company will not be available for all patients. A full economic evaluation including a societal costs and economic modelling is outside the scope of this research project.

## Conclusion

This study will provide information as to whether the integration of a non-dispensing pharmacist in primary care will improve medication safety compared to current care models.

## Abbreviations

HARM: Hospital Admission Related to Medication
GP: General Practitioner
NDP: Non-Dispensing Pharmacist
POINT: Pharmacotherapy Optimisation through Integration of a Non-dispensing pharmacist in a primary care Team
CPTC: Clinical Pharmacy Training Program
